# Craniofacial Diseases Caused by Defects in Intracellular Trafficking

**DOI:** 10.3390/genes12050726

**Published:** 2021-05-13

**Authors:** Chung-Ling Lu, Jinoh Kim

**Affiliations:** Department of Biomedical Sciences, College of Veterinary Medicine, Iowa State University, Ames, IA 50011, USA; cllu@iastate.edu

**Keywords:** craniofacial diseases, intracellular trafficking, secretory pathway, endosome/lysosome targeting, endocytosis

## Abstract

Cells use membrane-bound carriers to transport cargo molecules like membrane proteins and soluble proteins, to their destinations. Many signaling receptors and ligands are synthesized in the endoplasmic reticulum and are transported to their destinations through intracellular trafficking pathways. Some of the signaling molecules play a critical role in craniofacial morphogenesis. Not surprisingly, variants in the genes encoding intracellular trafficking machinery can cause craniofacial diseases. Despite the fundamental importance of the trafficking pathways in craniofacial morphogenesis, relatively less emphasis is placed on this topic, thus far. Here, we describe craniofacial diseases caused by lesions in the intracellular trafficking machinery and possible treatment strategies for such diseases.

## 1. Introduction

Craniofacial malformations are common birth defects that often manifest as part of a syndrome. These developmental defects are involved in three-fourths of all congenital defects in humans, affecting the development of the head, face, and neck [1]. Overt craniofacial malformations include cleft lip with or without cleft palate (CL/P), cleft palate alone (CP), craniosynostosis, microtia, and hemifacial macrosomia, although craniofacial dysmorphism is also common [2]. Disease-causing variants are found in many genes, and the implicated genes are involved in diverse cellular processes. During craniofacial developmental processes, many receptors and ligands are orchestrated to produce the appropriate cellular signaling necessary for proper craniofacial formation [3]. These molecules are synthesized in the endoplasmic reticulum (ER) and are transported to their destinations through intracellular trafficking pathways. Therefore, alterations in trafficking of these signaling molecules can lead to craniofacial malformation.

**Figure 1:**
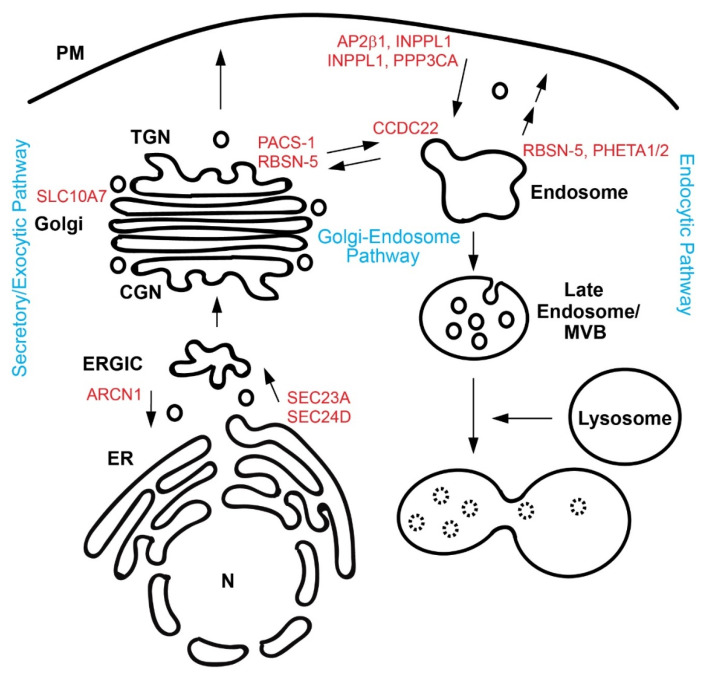
Schematic diagram of intracellular trafficking pathways. Genes/Proteins involved in craniofacial malformation are shown in red and are positioned in the place where they are believed to function. Three trafficking pathways are shown in blue. CGN, *cis*-Golgi network; ER, endoplasmic reticulum; ERGIC, ER-Golgi intermediate compartment; MVB, multi-vesicular body; N, nucleus; PM, plasma membrane; and TGN, *trans*-Golgi network.

Cells use membrane-bound carriers to transport such signaling molecules. The intracellular trafficking pathways include the secretory pathway, Golgi-endosome/lysosome pathways, and endocytic pathways (Figure 1). The secretary pathway is responsible for exporting nearly one-third of newly synthesized proteins from the ER to the Golgi complexes, and finally to the cell surface/extracellular space [4]. Cargo proteins are often post-translationally modified during this journey [5]. Golgi-endosome/lysosome pathways can deliver newly synthesized proteins to endosomes and lysosomes, and can retrieve cargo molecules, such as sorting receptors, bringing them back to the Golgi [6]. Cells can internalize cell surface receptors and ligands by endocytosis [7]. Endocytic vesicles generated from the plasma membrane (PM) fuse with recycling endosomes, where sorting decisions take place, resulting in recycling of cargo to the PM or eventual degradation of cargo in lysosomes [8].

In addition to the signaling ligands and receptors, the extracellular matrix (ECM) plays a critical role in craniofacial development, as the ECM serves as structural support and a protein-binding platform for the signaling molecules [9]. The ECM is composed of glycoproteins, collagens, glycosaminoglycans, and proteoglycans. ECM components are synthesized in the ER and secreted through the secretory pathway [4]. Therefore, a defect in the secretory pathway can alter the distribution of ECM components, resulting in the aberrant signaling and craniofacial abnormality. In this work, we review how intracellular trafficking pathways are implicated in craniofacial diseases.

## 2. Craniofacial Diseases Arising from Intracellular Trafficking Defects

### 2.1. The Secretory/Exocytic Pathway

#### 2.1.1. SEC23A

SEC23A is a component of coat protein complex II (COPII). COPII proteins are comprised of SAR1, SEC23/SEC24, and SEC13/SEC31. COPII vesicles deliver cargos from the ER to the ER-Golgi intermediate compartment in mammalian cells [10]. SEC23 is the GTPase activating protein for SAR1 and also recruits SEC13/31 to the ER exit sites. Mutations in *SEC23* disrupt the COPII vesicle assembly, resulting in accumulation of cargos in the ER. A novel dysmorphic syndrome, cranio-lenticulo-sutural dysplasia (CLSD), was described in an inbred Saudi Arabian family (see Table 1 for detail) [11]. Affected individuals have wide-open and late-closing fontanels and Y-shaped sutural cataracts. A highly conserved homozygous variant (F382L) was identified in *SEC23A* and this variant was found in the SEC31-binding groove [12]. Fibroblasts derived from a CLSD patient showed distended ER and a significant accumulation of type I collagen in the ER [12]. Zebrafish *crusher* mutants having mutations in *sec23a* showed craniofacial cartilage defects and a secretion block of ECM components in chondrocytes [12]. Another patient showing CLSD-like phenotypes was identified with a heterozygous *SEC23A M702V* variant, which is also located in the SEC31-binding groove [13]. Type I collagen accumulated in the ER, and ER distension was observed in the patient’s fibroblasts.

#### 2.1.2. SEC24D

SEC24 forms a complex with SEC23. SEC24 is mainly responsible for sorting cargos into nascent COPII vesicles, and *SEC24* mutations disrupt cargo sorting [14]. Individuals with compound heterozygous variants in *SEC24D* (e.g., S1015F and Q205*, S1015F and Q978P, and R484* and R313H) showed a large fronto-parietal apical ossification defect of the skull, and fractures in long bones [15,16,17] (Table 1). S1015 is located near a cargo-binding pocket and SEC24D S1015F is defective in cargo sorting [15,18]. Fibroblasts derived from a patient showed a mild accumulation of type I collagen in the ER and dilated ER. *sec24d* knockout mutants of zebrafish and medaka recapitulated human phenotypes, and showed a strong accumulation of type II collagen in chondrocytes [19,20].

#### 2.1.3. Archain 1 (ARCN1)

The coat protein complex I (COPI) operates in Golgi-to-ER and intra-Golgi transport [21]. COPI is a heptametric protein complex composed of α-COP, β-COP, β’-COP, γ-COP, δ-COP, ε-COP, and ζ-COPI [21]. Izumi et al. reported a genetic disorder caused by loss-of-function variants in *ARCN1*, which encodes for δ-COP [22]. Affected individuals presented with severe micrognathia, rhizomelic shortening, microcephalic dwarfism, and mild developmental delay (Table 1) [22]. The variants led to a reduction in *ARCN1* mRNA levels. Knocking down *ARCN1* expression caused the accumulation of type I collagen and triggered ER stress in cells. *nur17* mice with a homozygous missense variant in exon 10 of *Arcn1* showed neurological defects and low body weight, consistent with human phenotypes [23]. However, *nur17* mice did not present with skeleton phenotypes. The authors postulated that this was due to mild loss-of-function effects of the mouse variant [23].

#### 2.1.4. Solute Carrier Family 10, Member 7 (SLC10A7)

A combined approach of glycomics and whole-exome sequencing revealed *SLC10A7* as a causal gene in patients with congenital disorders of glycosylation [24]. Ashikov et al. reported that patients with SLC10A7 deficiency show short stature, defective enamel formation (amelogenesis imperfecta), skeletal dysplasia, facial dysmorphism, moderate hearing impairment, and mildly impaired intellectual development (Table 1). Zebrafish *slc10a7* morphants showed similar phenotypes [24]. *slc10a7* mutant mice also showed similar craniofacial and skeletal defects [25]. SLC10A7 is implicated in Golgi homeostasis and glycoprotein trafficking [24]. SLC10A7 is located in the Golgi, and cells derived from a patient showed a partial accumulation of sialylated glycol-conjugates in early endosomes [24].

### 2.2. Golgi-Endosomes/Lysosome Pathways

#### 2.2.1. Phosphofurin Acidic Cluster Sorting Protein-1 (PACS-1)

PACSs are members of cytosolic connector proteins. PACS-1 is responsible for locating furin and mannose-6-phosphate receptors to the trans-Golgi network, and thus, plays an important role in protein sorting at trans-Golgi network [26]. An exome sequencing identified a de novo variant in *PACS-1* from two unrelated patients who share similar facial dysmorphisms, including hypertelorism with down slanting palpebral fissures, mild synophrys with highly arched eyebrows, long eyelashes, down turned corners of the mouth, a thin narrow upper lip, and intellectual delay (Table 1) [27]. An expression of mutant *PACS-1* mRNA in zebrafish embryos induced craniofacial defects in a dominant-negative fashion [27].

#### 2.2.2. Coiled-Coil Domain-Containing Protein 22 (CCDC22)

CCDC22 forms a complex with copper metabolism MURR1 domain-containing 1. This complex can regulate copper deprivation through retrograde transport of ATP7B from peripheral endosomes back to the trans-Golgi network [28]. A missense variant in CCDC22 causes Ritscher-Schinzel (RSS) syndrome, with common facial signs including a broad forehead, up slanting palpebral fissures, wideset eyes, a short philtrum, and a broad neck with a low posterior hair line (Table 1 and Table 2) [29]. This disease is an X-linked recessive disorder. Patients with RSS syndrome have 50% lower CCDC22 expression than controls [29,30].

### 2.3. Endocytic Pathways

#### 2.3.1. Adaptor-Related Protein Complex 2, β-1 Subunit (AP2β1)

Adaptor protein complexes sort cargos during clathrin-coated vesicle assembly [31]. The adaptor protein complexes include adaptin proteins (α, γ, δ, or ε and β1, β2, or β4). AP2β1 complex is important for clathrin-dependent endocytosis from the PM [32]. An insertional mutation within the *Ap2β1* gene causes non-syndromic cleft palate in mice, which is inherited in an autosomal recessive manner [33]. Homozygous mutant embryos lacked the *Ap2β1* gene expression. The mechanism through which the *Ap2β1* mutation causes the cleft palate is still unclear. Li et al. proposed that the lack of the β-adaptin might cause defective internalization of receptors or cell surface proteins involved in palatogenesis [33]. As TGF-β signaling pathway plays a critical role in palatogenesis [34], the authors suggested that the cleft palate phenotype of β2-adaptin-defecient mice results from the disruption of TGF-β internalization [33]. However, it remains to be determined if indeed the TGF-β internalization defect is observed in the mutant mice and is responsible for the phenotype.

#### 2.3.2. Inositol Polyphosphate Phosphatase-Like 1 (INPPL1)

Below et al. identified variants in the *INPPL1* gene from patients with opsismodysplasia [35]. The patients presented with short limbs, and small hands and feet, relative macrocephaly with a large anterior fontanel, and characteristic craniofacial abnormalities (Table 1) [35]. Six out of twelve variants resulted in premature stop codons, two out of twelve variants were splice-site variants, and four out of twelve variants were missense variants in the catalytic domain [36]. INPPL1 plays a crucial role in the endocytosis of Ephrin (EPH) receptors. Thus, cell surface levels of the EPH receptors might be dysregulated by the INPPL1 variants [37].

#### 2.3.3. PH Domain-Containing Endocytic Trafficking Adaptor 1 and 2 (PHETA1/2, a.k.a. FAM109A/B, SES1/2, IPIP27A/B)

PHETA1 and 2 provide a binding site for inositol polyphosphate–phosphatase, OCRL, which is the causal gene for oculocerebrorenal syndrome of Lowe [38]. OCRL interacts with several endocytic proteins to regulate recycling of receptors at sorting and recycling endosomes [38,39]. Thus, PHETA1 and 2 likely play a role in endocytic trafficking. PHETA1and 2 are also involved in the sorting of lysosomal hydrolases [38]. A patient with craniofacial dysmorphism was identified with a heterozygous de novo arginine to cysteine variant (R6C) in *PHETA1* (Table 1) [40]. As the variant did not affect its mRNA expression, the functional role of PHETA1 and PHETA2 was analyzed in zebrafish mutants. Cranial distance (the distance between the most anterior Meckel’s cartilage and lateral fins), ceratohyal distance (the distance between ceratohyal cartilage joints and lateral fins), ceratohyal length, Meckel’s area, jaw width, and jaw length were measured to describe craniofacial phenotypes in zebrafish [41]. *pheta 2* knockout mutants showed more severe craniofacial phenotypes than *pheta 1* knockout mutants. In *pheta* 1 knockout animals, Meckel’s area and the width and length of the jaw were reduced, but *pheta* 2 knockout animals were more severely affected in all parameters measured [40]. Interestingly, the patient with the de novo heterozygous R6C variant in *PHETA1* had a significantly reduced jaw width [40]. As PHETA1 and 2 can form both a homodimer and a heterodimer, it was suggested that the R6C variant acts on the craniofacial development in a dominant-negative manner.

#### 2.3.4. Calcineurin (PPP3CA)

PPP3CA is activated upon calcium influx and dephosphorylates endocytic proteins to control the speed of endocytosis in secretory cells [42,43]. Individuals were found with de novo variants (Ala473Thr; Phe470Leu) in the catalytic domain and the auto-inhibitory (AI) domain of PPP3CA [44]. Patients with defects in the catalytic domain showed non-syndromic epileptic encephalopathy with spasms and hypsarrhythmia, leading to a clinical diagnosis of West syndrome (Table 1 and Table 2). Patients with defects in the AI domain showed developmental delay with seizure and dysmorphic features including trigonocephaly, cleft palate, micrognathia, brachydactyly, arthrogryposis, and a short stature [44].

#### 2.3.5. Rabenosyn-5 (RBSN-5)

RBSN-5 contributes to the recycling of transferrin receptors to the PM and the trafficking of cathepsin D from the Golgi to lysosomes. A patient with a homozygous missense variant (Gly425Arg) in *RBSN-5* presented with intractable seizures, developmental delay, microcephaly, dysostosis, osteopenia, craniofacial dysmorphism, macrocytosis, and megaloblastoid erythropoiesis (Table 1) [45]. The Gly425Arg missense variant did not affect the expression or localization of RBSN-5, but caused 50% reduction of transferrin levels in a patient’s cells [45]. In addition, cathepsin D remained in the Golgi, and its activity was reduced to 35% in the patient’s fibroblasts [46].

### 2.4. Remaining Questions

There remain many important questions related to the contribution of the trafficking pathways to craniofacial morphogenesis. For example, are there additional trafficking pathways that are implicated in craniofacial diseases? How does a variant protein dysregulate intracellular trafficking? How does such trafficking defect affect craniofacial formation? Does a trafficking defect of a particular cargo molecule or multiple cargo molecules account for respective phenotypes? How can such diseases be treated? As we answer these questions, we will be able to better understand the intimacy between intracellular trafficking and craniofacial morphogenesis.

SEC24 paralogs, which play a critical role in cargo sorting during ER export, might provide insight into some of those questions. There are four paralogs of SEC24 (A to D) in mammals and each has multiple, different cargo binding pockets [4]. A cargo binding pocket can interact with a specific ER export signal from a cargo protein. SEC24 paralogs can be categorized into two sub-groups (SEC24A/SEC24B and SEC24C/SEC24D), according to their sequence similarity and cargo preferences [5]. In addition, the expression profiles of *SEC24* genes vary in different tissues. Thus, both the cargo preferences and the expression profiles of SEC24 paralogs can affect the phenotypes of a defect from a particular SEC24 paralog. For example, *Sec24a* knockout mice developed and lived normally, but exhibited markedly reduced plasma cholesterol levels due to the ER export defect of PCSK9, a negative regulator of the low-density lipoprotein receptor [47]. *Sec24b* deletion caused a completely open neural tube in mice, which occurs because VANGL2 fails to be sorted into COPII vesicles [48]. Thus, these two cases can be explained by ER export defects of a particular cargo molecule. Compound heterozygous or homozygous *SEC24D* variants cause craniofacial and skeletal defects in humans and zebrafish [15,19,20]. ER export defects of collagen partly account for the bone defects. However, other cargo molecules might contribute to disease phenotypes. For example, matrilin accumulated in the ER in *sec24d* knockout zebrafish mutants [20]. In mice, homozygous *Sec24d* knockout mutants were embryonic lethal before bone development [49], clearly indicating that SEC24D plays a crucial role in other developmental pathways. Humans or animals having a heterozygous *SEC24D* variant were indistinguishable from wild-type counterparts. Interestingly, a much more severe collagen secretion defect was observed in the yolk sac than in the embryo of *Sec24d* knockout mice. This tissue-specific phenotype was correlated with the absence of SEC24B in the yolk sac [50]. Thus, specific cargo molecules relevant to the phenotypes of a disease and tissue-specific expression profiles of SEC24 paralogs allow us to better understand the pathology of the disease.

### 2.5. Therapeutic Strategies

Since the patients described above do not have defects in cargo molecules, this presents a unique opportunity for treatment strategies, namely that there might be ways to restore trafficking of such cargo molecules. One possibility is to turn on the expression of a paralog(s). If there is a paralog(s) for a mutated gene and the paralog happens to not be expressed in the affected tissue, expressing such a paralog would restore cargo trafficking. A small molecule or a CRISPR technology (see below for CRISPRa) that could stimulate the paralog expression could be a treatment option. In the case of diseases involving ER export failure, chemicals enhancing proteostasis could be considered. For example, 4-phenylbutyrate (4-PBA) promotes trafficking of a broad range of membrane proteins and secretory proteins [51]. Although 4-PBA was an FDA-approved drug for management of urea cycle disorders, it was discovered that 4-PBA could target SEC24 and enhance ER export of cargo molecules [52]. 4-PBA treatments in humans were conducted in adults having cystic fibrosis caused by a ΔF508 CFTR (cystic fibrosis transmembrane conductance regulator) variant, and it led to the partial restoration of chloride transport in the nasal epithelium, with a specific recommended dose range [53]. However, it remains to be determined if it could be used for fetuses and infants. CRISPR-mediated activation (CRISPRa) is another therapeutic option for stimulating gene expression (e.g., paralog expression) [54]. Matharu et al. showed that CRISPRa can target endogenous gene regulators (promoter or enhancer) to increase the expression of *Sim1*, resulting in a rescue of the obesity phenotype in a *Sim1* haploinsufficient mouse model [55].

Enzyme replacement therapy (ERT) has been used to treat lysosomal storage diseases. ERT can replace defective lysosomal enzymes with infusion of recombinant human enzymes, resulting in restoration of the enzymatic activity in patient lysosomes [56]. However, caveats are that most infused recombinant enzymes seem to be delivered to the visceral organs, such as the liver, kidney, and spleen, and the infused enzymes rarely cross the blood–brain barrier [57,58,59]. Even though ERT is mostly restricted to adults, a study showed that infants with genetic disorders could be treated using ERT with a recommended dose [60].

Ideally, gene therapy can cure genetic diseases. Gene therapy introduces foreign gene elements into a host genome, using an appropriate vector like adeno-associated virus vectors [61]. Clinical trials of gene therapy are in use for an increasing number of genetic diseases. A gene editing tool, CRISPR-Cas9, has been used for a treatment of human genetic diseases. For example, CRISPR-Cas9 was used in people with a severe form of a genetic disorder called β-thalassaemia. Patients who received the treatment did not need blood transfusion [62,63].

## 3. Conclusions

Advanced understanding of intracellular protein trafficking is necessary for designing novel and improved treatment strategies for the diseases described in this review. It seems that we are scratching the surface of the connection between craniofacial morphogenesis and intracellular trafficking. As we expect to discover more diseases in this category in the future, this connection will become increasingly more prominent. We hope that efforts from basic studies on intracellular trafficking and a multitude of therapeutic strategies yield viable treatments for these diseases.

## Figures and Tables

**Table 1:** Genes in intracellular trafficking pathways linked to craniofacial diseases.

Gene	Craniofacial Features	Extra Cranial Features
*Sec23A*	Wide-open calvarial sutures with large and late-closing anterior fontanels, frontal bossing, hyperpigmentation with capillary hemangioma of the forehead, significant hypertelorism, a broad and prominent nose, and Y-shaped sutural cataracts	Short stature, coarse brittle and scarce hair, dorsal wedging of the vertebral bodies, and high and narrow iliac wings
*Sec24D*	Ocular proptosis with orbital craniosynostosis, hydrocephalus, frontal bossing, midface hypoplasia, and micrognathia	Multiple bone fractures, usually resulting from minimal trauma, bone deformity
*ARCN1*	Severe micrognathia and microcephalic dwarfism.	Rhizomelic shortening and mild developmental delay.
*SCL10A7*	Defective enamel formation (amelogenesis imperfecta), coarse/dysmorphic face, teeth anomalies, and mandibular hypoplasia	Short stature, chest deformity, moderate hearing impairment, and mildly impaired intellectual development
*PACS-1*	Hypertelorism with down slanting palpebral fissures, mild synophrys with highly arched eyebrows, long eyelashes, downturned corners of the mouth, and a thin narrow upper lip	Intellectual delay
*CCDC22*	Ritscher-Schinzel syndrome (RSS, see Table 2), broad forehead, up slanting palpebral fissures, wideset eyes, a short philtrum, and a broad neck with a low posterior hair line	Ventricular septal defect and Dandy-Walker syndrome (see Table 2)
*AP2β1*	Cleft palate	
*INPPL1*	Opsismodysplasia, relative macrocephaly with a large anterior fontanel, hypertelorism, high forehead, short nose, long philtrum, large fontanelle, coarse face, mid face hypoplasia, and brachycephaly	Short limbs, and small hands and feet
*PHETA1/2*	Facial asymmetry, coarse facial features, concave nasal ridge, flat occiput, malar flattening, narrow mouth, sparse scalp hair, relative macrocephaly, abnormality of dental morphology, and widely spaced teeth	Scoliosis, clinodactyly of fourth and fifth digits on both hands, multiple palmar and planar creases, pes planus, short foot and palm, tapered fingers, slow-growing nails, and metatarsus adductus
*PPP3CA*	Trigonocephaly, cleft palate, and micrognathia	West syndrome (see Table 2), brachydactyly, a short stature, and arthrogryposis
*RBSN-5*	Microcephaly, midfacial bone hypoplasia, deep-set eyes with a hooded appearance, a fullness in the nasal bridge, short nose, and a large mouth with small teeth and tongue protrusion	Developmental delay, macrocytosis, megaloblastoid erythropoiesis, moderate osteopenia involving the pelvis and long bones of both upper and lower limbs, with evidence of undertubulation and hypoplasia of the epiphyses around the knee joint and bilateral coxa valga

**Table 2:** Features of the syndromes described in this work.

Syndrome	Craniofacial Features	Extracranial Features
Dandy-Walker syndrome		Enlargement of the fourth ventricle (a small channel that allows fluid to flow freely between the upper and lower areas of the brain and spinal cord), a partial or complete absence of the cerebellar vermis (the area between the two cerebellar hemispheres), and cyst formation near the internal base of the skull
West syndrome		Axial spasms, psychomotor retardation, and a hypsarrhythmic interictal electroencephalopathy pattern
Ritscher-Schinzel syndrome	Macrocephaly, a prominent forehead and occiput, foramina parietalia, hypertelorism, down slanting palpebral fissures, depressed nasal bridge, narrow palate, and apparently low-set ears	Communicating hydrocephalus, aplasia of the posterior portion of the cerebellar vermis, and high insertion of the confluent sinus
